# Hyperglycaemia in critically ill patients: the immune system’s sweet tooth

**DOI:** 10.1186/s13054-017-1775-1

**Published:** 2017-08-03

**Authors:** Gustav van Niekerk, Tanja Davis, Anna-Mart Engelbrecht

**Affiliations:** Department of Physiological Sciences, Stellenbosch University, Private Bag X1, Matieland, Stellenbosch 7600 South Africa

**Keywords:** Autophagy, Glycaemic control, Hyperglycaemia, Immunometabolism, Insulin, Critical Illness, Sepsis, Glycolysis, Metabolism

## Abstract

There is an ongoing debate regarding the efficacy of glycaemic control in critically ill patients. Here we briefly highlight the key function of elevated glucose in critically ill patients, namely, to enable elevation of aerobic glycolysis in rapidly dividing cells. In particular, aerobic glycolysis provides metabolic intermediates necessary for expansion of biomass in immune cells and promotion of tissue repair. Furthermore, we emphasise that insulin may inhibit autophagy, a cell survival process used in the bulk degradation of cellular debris and damaged organelles. These observations provide a rational basis for tolerating elevated glucose levels in certain critically ill patients.

## Introduction

The hyperglycaemia observed during immune activation was initially seen as an adaptive response linked to the normal stress response. This view was challenged by the findings that insulin therapy in critically ill patients to reduce glucose levels below 110 mg/dL (6.1 mmol) resulted in a dramatic decrease in mortality [1]. Subsequently, the hyperglycaemia observed during an infection has been described as a defect of glucose homeostasis [2]—a manifestation of pathology that must be treated. A number of observations also support this view. As an example, hyperglycaemia has consistently been associated with an adverse outcome in patients with sepsis, irrespective of their diabetic status [3]. Furthermore, hyperglycaemia is not only associated with activation of inflammatory responses, but in fact has been found to be a causative agent behind the development of certain pathologies. This is particularly well exemplified by literature on the consequence of hyperglycaemia observed in diabetes. In diabetic patients, continual hyperglycaemia can injure endothelial cells and decrease the capillary bed density in various organs. Mechanistically, hyperglycaemia can increase the formation of advanced glycation end products (AGE) when proteins and lipids become glycated. This may promote a number of detrimental consequences, particularly the binding of AGE to the receptors for advanced glycation end products (RAGE) on immune cells, which may illicit an inflammatory response [4]. There is also evidence that hyperglycaemia might cause mitochondrial damage. In mice, prolonged exposure to an obesogenic diet resulted in insulin resistance, followed by mitochondrial dysfunction associated with an increase in reactive oxygen species production [5]. These changes were also observed when diabetes was chemically induced (treated with streptozotocin), suggesting that increased mitochondrial dysfunction was a result of hyper-glycaemia/lipidaemia [5]. These observations provide a mechanistic foundation by which hyperglycaemia adversely affects critically ill patients, thus providing a rational basis for treating hyperglycaemia in critically ill patients.

However, a number of subsequent studies have failed to show the benefits of glycaemic control [6–11], and some have even found adverse effects associated with strict glycaemic control [12]. The Surviving Sepsis Campaign now recommends targeting blood glucose levels below 180 mg/dL instead of the previous tight (<110 mg/dL) glycaemic levels [13]. Hence, the targeting of glucose levels remains a topic that solicits intense debate [14–16]. What is evident, however, is that some patients do seem to benefit from controlled glucose levels, whereas others obviously do not.

A growing understanding of the metabolic needs of activated immune cells provides a new view on the effect of glycaemic control. Here we briefly review both established work as well as recently emerging insights into the metabolism of activated immune cells. It is argued that glycaemic control might in effect exert an immune-suppressive effect, which may diminish the host’s ability to launch a competent immune response. In fact, the anti-inflammatory effects of insulin might mediate some of the beneficial effects of glycaemic control, suggesting that the metabolism of immune cells may represent novel targets for anti-inflammatory interventions. Secondly, insulin itself may adversely affect patient outcome by inhibiting autophagy, a generic cell survival response. From these considerations, it is clear that glycaemic control may not represent optimum support for all patients.

## The metabolic sweet spot

Lactic acidosis has been ascribed to systemic anaerobic conditions resulting from hypoperfusion manifesting as tissue hypoxia. Indeed, sepsis is often associated with a decrease in blood pressure (in severe cases manifesting as septic shock) which could leave tissue hypoxic. However, evidence emerged that lactic acid build-up poorly correlates with tissue hypoxia. It has been shown that sepsis does not compromise oxygen delivery compared to normal individuals [17]. Indeed, therapies aimed at increasing oxygenation failed to improve clinical parameters [18, 19]. These observations suggest that lactate build-up is not solely dependent on tissue hypoxia [18, 19]. Other factors may include mitochondrial dysfunction resulting from severe inflammation [20]. In addition, an increase in lactate production by normal cells may also contribute. Indeed, numerous cells make use of ‘aerobic glycolysis’—the lactic acid fermentation of sugar despite the abundance of oxygen. Vascular endothelium cells, despite being in direct contact with oxygenated blood, presumably spare the oxygen supply on route for intended tissue [21]. Cancer cells also exhibit an increase in glycolysis despite the presence of oxygen—a phenomena referred to as the ‘Warburg effect’ (named after Otto Warburg who first described aerobic glycolysis in cancerous cells). Aerobic glycolysis is not unique to proliferating cancer cells, but is also a normal metabolic strategy enacted by various rapidly dividing cells [22], including immune cells [23]. Thus, an increase in aerobic glycolysis conducted by activated immune cells likely contributes to the increase in lactic acid build up through glycolytic activity in other tissues, as well as a decrease in lactic acid clearance.

Yet, anaerobic glycolysis is highly inefficient: whereas oxidative respiration yields 32 moles of ATP for each mole of glucose fully oxidised, glycolysis only produces 2 moles of ATP. This then raises the question why the immune system would make use of such a seemingly ineffective system. One key benefit of making use of aerobic glycolysis relates to energy production: though the *efficiency* of ATP might be lower for aerobic glycolysis compared to oxidative phosphorylation, the *rate* at which ATP can be produced is much higher (i.e. more ATP can be produced by glycolysis than oxidative phosphorylation in a given time unit) [24]. This would suggest that elevated glycolysis in immune cells might represent a metabolic strategy to rapidly increase cellular ATP levels.

Energy production is not the only endpoint of aerobic glycolysis in rapidly dividing cells. Indeed, another function of glycolysis is to provide metabolic intermediates used in other biosynthetic pathways, such as for the synthesis of lipids and nucleotides [22]. This also explains why, in several cancer types, the contribution of glycolysis to ATP production is marginal despite high glucose consumption [25]. The application of aerobic glycolysis is now also understood to play a pivotal role in the activated immune cells of both the innate and adaptive immune systems [26, 27]. As an example, activated monocytes rapidly increase the biosynthesis of fatty acids [28]. Interestingly, following inhibition of fatty acid synthesis with RNA interference, markers of macrophage differentiation were decreased [28], indicating the reliance of differentiation on metabolism. Here, glycolysis can be indispensable in providing the metabolic intermediates (such as acyl-CoA) which can be used for lipid synthesis [22]. The use of glucose for biosynthetic processes is similarly important in cells of the adaptive immune system. As an example, upon activation of a corresponding antigen, B cells rapidly upregulate glucose uptake and glycolysis [29]. Moreover, upregulation of the pentose phosphate pathway (PPP) prior to cells entering the S phase was also observed. This observation suggests that glucose might be shifted towards biosynthetic pathways, since the PPP is also implemented to provide metabolic intermediates [29].

Taken together, it is clear that glucose plays a central role in the functioning of activated immune cells. Glucose is important for both energy production and maintaining biosynthetic activities associated with the rapid expansion of immune cells and the production of immune modulators/effectors during an infection. This also suggests that hampering glucose supply would likely adversely affect immune cell function.

## Addressing the immunological needs: hyperglycaemia

It is thus pivotal that immune cells receive adequate amounts of glucose. Indeed, energy production by glycolysis can only out-perform oxidative phosphorylation under conditions of high glucose uptake [30]. Similarly, low glucose levels are likely to compromise cellular biosynthetic capacities. In this regard, a number of physiological adaptations exist to augment the glucose supply chain. Firstly, activated immune cells rapidly upregulate the expression of glucose transporters [31], thus enhancing the rate at which glucose is imported. Interestingly, it has also been noted that insulin plays an important role in T cells, since T cells lacking insulin receptors exhibit a dramatically reduced glycolytic capacity [32]. This is surprising since insulin levels are usually normal or slightly suppressed during sepsis [33]. Regardless, glucose transporters follow Michaelis–Menten kinetics, which implies that substrate concentration (i.e. serum glucose levels) will influence the rate at which glucose is transported into cells.

**Fig. 1:**
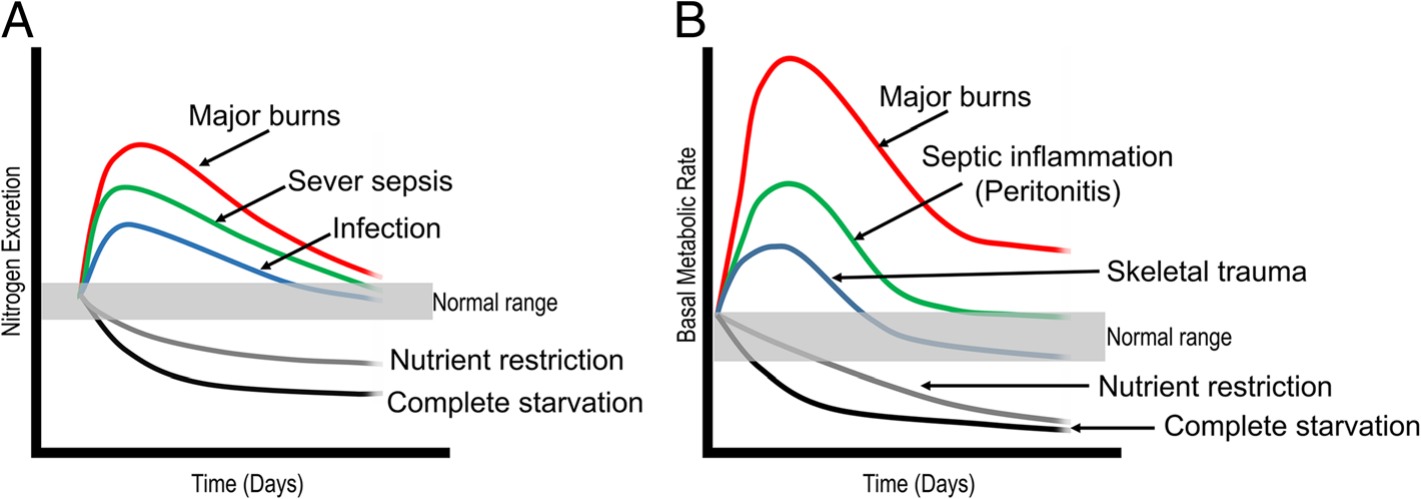
Activation of an immune response is associated with an increase in metabolic turnover **a** sustained by increased protein catabolism (**b**). Redrawn with modification from [48]

Serum glucose levels are elevated through a range of physiological mechanisms. Various inflammatory mediators, such as Il-1b and TNF [34], Il-6 [35], as well as type I and II interferons [36], induce insulin resistance. In addition, evidence from mouse models suggests that a decrease in blood flow to muscle might also contribute to the lower glucose consumption in response to a lipopolysaccharide challenge [37]. However, gluconeogenesis in the liver is a major contributing factor towards the development of hyperglycaemia [2]. In fact, an increase in nitrogen secretion reflects the increase in basal metabolic rate (Fig. 1), as the carbon skeleton of amino acids is used to produce glucose, which in turn fuels the elevated metabolic state. Mechanistically, inflammatory cytokines such as Il-6 increase the secretion of glucagon by acting both on the central nervous system as well as directly on islets cells [38]. Taken together, these responses demonstrate the physiological adaptation to the unique metabolic needs of immune cells during an infection, and that altered glucose metabolism during an infection does not represent a ‘dysregulation’ but a tailored response geared towards the mobilisation of an effective immune response.

These arguments suggest that hyperglycaemia is in service of promoting and sustaining a competent immune response. However, this does not mean that an immune response is necessarily adaptive or desirable. Strict glycaemic control might exert beneficial effects by actually impeding immune function. This might explain why certain patients do benefit from insulin therapy. It is worth noting that the Leuven study was predominantly in patients that were assigned to an ICU after cardiac surgery [1]. In the context of such sterile intervention, attenuating immune function by glycaemic control might be beneficial in avoiding unnecessary inflammation. In contrast, inhibiting immune function in the context of an infection could be disastrous.

## Insulin and autophagy

Insulin, independent of altering glucose levels, may also have unanticipated adverse effects. It has long been noted that an anabolic hormone such as insulin can inhibit the catabolic process of autophagy [39]. In fact, evidence suggests that critically ill patients not only exhibit traits of autophagic insufficiency, but that these effects can be exacerbated by insulin therapy: hepatocytes from critically ill patients receiving insulin as part of glucose control exhibited an 80% greater reduction in autophagic vacuole formation compared to critically ill patients receiving standard therapy [40]. Such an inhibition of autophagy may have several adverse effects since autophagy plays a pivotal role in both host defence as well as cell survival [41]. In host defence, autophagy plays a key function in the processing of epitopes to be loaded on both MHC I and II, targeting intracellular bacteria for autophagy degradation (referred to as xenophagy). Furthermore, vesicles used for viral replication are also degraded via autophagy. These processes are operative not only in immune cells, but also in non-immune cells, highlighting the potential role of maintaining elevated levels of autophagy during an infection [41].

Autophagy may also play a key role in surviving the adverse conditions faced in a clinical setting, including the removal of damaged or misfolded proteins (aggrephagy) as well as damaged mitochondria (mitophagy) or the clearance of noxious factors such as bacteria and endotoxins by the liver [41]. As an example, critically ill patients demonstrate mitochondrial dysfunction with a concomitant decrease in antioxidant capacity [42]. It is very likely that damaged mitochondria result in an increased formation of radicals, which in turn results in decreased antioxidant capacity. Here, autophagy plays a pivotal role in targeting damaged mitochondria for degradation [43]. The key role played by autophagy thus also raises concerns regarding the role of insulin in controlling hypoglycaemia while similarly also inhibiting autophagy. In this study, however, patients were in a fed state, which represents a confounding factor since feeding may suppress autophagy.

## Glycaemic control: novel therapeutic strategies

Activation of an inflammatory response is often accompanied by a decrease in appetite. Yet, despite a decrease in nutrients, rapid expansion of the immune system, as well as the synthesis of immune effectors, must be sustained. In this regard, catabolism in peripheral tissue is directed at maintaining anabolism of the immune system. Here we have argued not merely that hyperglycaemia during immune activation represents an evolutionarily conserved response, but have, in fact, pointed out the reason why elevated glucose levels may in fact play an important function during an infection. However, the manner in which metabolic alteration during an inflammatory insult promotes immune function remains to be formally investigated.

There is also a need to establish safe tolerable levels of glycaemic control as extreme levels of hyperglycaemia can, in fact, have a negative impact on immune function. In a rabbit model of critical illness, maintaining glucoses levels at 13.8–16.6 mmol/L and 13.875–19.425 mmol/L adversely affected innate immune cell activity [44, 45]. Similarly, an increase in glucose levels might be adaptive in the short term, but may exert negative effects (e.g. mitochondrial dysfunction [5]) in a chronic setting. This would suggest that glycaemic control might be beneficial in managing a protracted state. A key question remains whether glycaemic control might represent a strategy for attenuating unwanted immune activation. In this regard, insulin therapy is able to attenuate inflammation dramatically [46]. If such an immune-suppressive effect is indeed mediated by starving immune cells of glucose, it raises the intriguing possibility that novel anti-inflammatory therapies might be developed through targeting of immune cell metabolism. As an example, one meta-analysis [47] has failed to demonstrate any benefit of glycaemic control in patients with sepsis, though a benefit was observed in other ICU settings. This does not, however, indicate that insulin therapy may not have beneficial effects in sepsis. In this regard, insulin therapy may attenuate a life-threatening inflammatory response. There is thus a need to identify individuals who may best benefit from glycaemic control.

Emerging evidence suggests a key role for autophagy within a clinical context. However, more studies are required to investigate the effect of insulin therapy on autophagy. In the study by Vanhorebeek [40] critically ill patients were in a fed state, which would attenuate autophagy, thus representing a confounding factor. Also, if lowering glucose levels prevents cellular damaged caused by elevated glucose levels, it might mitigate the need for higher autophagy. In addition, since autophagy is activated in response to energy stress, it is also possible that hyperglycaemia might attenuate autophagy. Given the potential role of autophagy, establishing the effect of glycaemic control could be of great clinical value. More research is urgently needed to provide a complete picture of the effect exerted by intensive insulin therapy on autophagy.
